# The midgut transcriptome of *Lutzomyia longipalpis*: comparative analysis of cDNA libraries from sugar-fed, blood-fed, post-digested and *Leishmania infantum chagasi*-infected sand flies

**DOI:** 10.1186/1471-2164-9-15

**Published:** 2008-01-14

**Authors:** Ryan C Jochim, Clarissa R Teixeira, Andre Laughinghouse, Jianbing Mu, Fabiano Oliveira, Regis B Gomes, Dia-Eldin Elnaiem, Jesus G Valenzuela

**Affiliations:** 1 Vector Molecular Biology Unit, Laboratory of Malaria and Vector Research, National Institute of Allergy and Infectious Diseases, National Institutes of Health, Rockville, MD, 20852, USA; 2 Uniformed Services University of the Health Sciences, Bethesda, MD, USA; 3 Entomology Section, LMVR, NIAID, NIH, Rockville, MD, 20852, USA; 4 Malaria Genomics Section, LMVR, NIAID, NIH, Rockville, MD, 20852, USA

## Abstract

**Background:**

In the life cycle of Leishmania within the alimentary canal of sand flies the parasites have to survive the hostile environment of blood meal digestion, escape the blood bolus and attach to the midgut epithelium before differentiating into the infective metacyclic stages. The molecular interactions between the Leishmania parasites and the gut of the sand fly are poorly understood. In the present work we sequenced five cDNA libraries constructed from midgut tissue from the sand fly *Lutzomyia longipalpis *and analyzed the transcripts present following sugar feeding, blood feeding and after the blood meal has been processed and excreted, both in the presence and absence of *Leishmania infantum chagasi*.

**Results:**

Comparative analysis of the transcripts from sugar-fed and blood-fed cDNA libraries resulted in the identification of transcripts differentially expressed during blood feeding. This included upregulated transcripts such as four distinct microvillar-like proteins (LuloMVP1, 2, 4 and 5), two peritrophin like proteins, a trypsin like protein (Lltryp1), two chymotrypsin like proteins (LuloChym1A and 2) and an unknown protein. Downregulated transcripts by blood feeding were a microvillar-like protein (LuloMVP3), a trypsin like protein (Lltryp2) and an astacin-like metalloprotease (LuloAstacin). Furthermore, a comparative analysis between blood-fed and *Leishmania *infected midgut cDNA libraries resulted in the identification of the transcripts that were differentially expressed due to the presence of Leishmania in the gut of the sand fly. This included down regulated transcripts such as four microvillar-like proteins (LuloMVP1,2, 4 and 5), a Chymotrypsin (LuloChym1A) and a carboxypeptidase (LuloCpepA1), among others. Upregulated midgut transcripts in the presence of Leishmania were a peritrophin like protein (LuloPer1), a trypsin-like protein (Lltryp2) and an unknown protein.

**Conclusion:**

This transcriptome analysis represents the largest set of sequence data reported from a specific sand fly tissue and provides further information of the transcripts present in the sand fly *Lutzomyia longipalpis*. This analysis provides the detailed information of molecules present in the midgut of this sand fly and the transcripts potentially modulated by blood feeding and by the presence of the Leishmania parasite. More importantly, this analysis suggests that *Leishmania infantum chagasi *alters the expression profile of certain midgut transcripts in the sand fly during blood meal digestion and that this modulation may be relevant for the survival and establishment of the parasite in the gut of the fly. Moreover, this analysis suggests that these changes may be occurring during the digestion of the blood meal and not afterwards.

## Background

Leishmaniasis is a spectrum of diseases caused by numerous species of the kinetoplastid parasite Leishmania which are transmitted by Phlebotomine sand flies. Different forms of disease presentation can be linked with the various species of Leishmania parasites, with the visceral form of the disease being caused mainly by the Old World *Leishmania infantum *or the New World variant *Leishmania infantum *(*chagasi*). Visceral leishmaniasis is a disease which is commonly fatal if left untreated. Currently, there is no licensed vaccine for the prevention of visceral disease in humans and current drug treatment with antimonials and other components is a lengthy and arduous procedure with undesirable secondary effects [1].

The sand fly *Lutzomyia longipalpis*, the principal vector of the parasite *Leishmania infantum chagasi*, is the most significant source of American visceral leishmaniasis. As with many other arthropod-borne diseases, transmission of the Leishmania parasite, occurs during the act of vector blood feeding upon a vertebrate host. Upon blood meal ingestion a large number of events are induced, including digestion, metabolism, diuresis, and ultimately oogenesis. Unlike arboviruses, Plasmodium or Borrelia, Leishmania can complete the necessary developmental changes and propagate to numbers sufficient for transmission and infection solely within the confines of the midgut tissue of the sand fly [2]. Several sand fly proteases involved in blood meal digestion and implicated in the species specificity between Leishmania and the respective vectors have been characterized and include trypsins, chymotrypsins and chitinases from both *L. longipalpis *and *Phlebotomus papatasi *[3,4].

More global approaches to identifying and characterizing sand fly molecules has been accomplished through the sequencing of whole sand fly-derived expressed sequence tags [5]. While that study contributes to the knowledge of the molecular components of the sand fly it does not provide the specific molecules of the midgut tissue, which would interact with the developing parasites. The construction and sequencing of midgut tissue-specific cDNA libraries aims therefore, to identify those molecules involved in blood meal digestion and metabolism, peritrophic matrix formation, and possible parasite associations. Here we have generated and sequenced five cDNA libraries from the midgut tissue from *L. longipalpis*; investigated the molecules present during sugar and blood feeding as well as after the blood meal has been processed and excreted, both in the presence and absence of *L. infantum chagasi*. In addition to the identification of midgut-associated molecules, sequence analysis and phylogenetic comparison of the sequences of *L. longipalpis *allows a better understanding of blood meal processing in sand flies and the differences between visceral (*Lutzomyia longipalpis*) and cutaneous leishmaniasis (*Phlebotomus papatasi*) sand fly vectors.

## Results and discussion

As the midgut is the primary organ of the sand fly in which the Leishmania parasite develops, cDNA libraries of the midgut tissue were constructed, sequenced and analyzed to investigate the molecules present which may have important interactions between these two organisms. In total, five cDNA libraries were constructed from the midgut tissue of female *L. longipalpis *during different conditions of feeding and digestion. These conditions included one library combining the midguts from sand flies allowed to feed on a sucrose solution (SF), a pool of midgut tissue from sand flies fully engorged from an artificial blood meal 1, 2, and 3 days post blood meal ingestion (BF), and a pool of midguts from gravid sand flies 5, 6, and 7 days post blood meal digestion (PBMD). The conditions chosen and the pooling of those times after blood meal ingestion allows better coverage of the most abundant molecules transcribed in the midgut as well as a comparison of the molecules present prior to blood feeding, while the blood bolus is present, during digestion of the blood meal, and after the blood byproducts have been excreted. Two cDNA libraries were constructed from the equivalent pools of time points after blood feeding in *L. longipalpis *midgut tissue from sand flies which had ingested amastigote-infected macrophages in an artificial blood meal (BFi and PBMDi), a more natural presentation of parasites to the blood-feeding sand fly.

**Figure 1:**
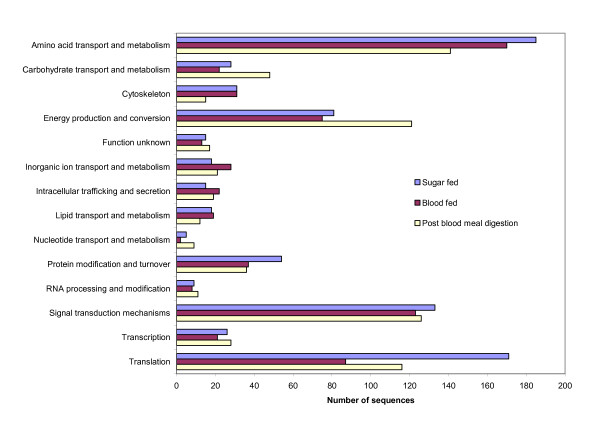
Histograph of the number of sequences grouped into functional classes from the sugar fed, blood fed and post blood meal digestion cDNA libraries. Sequences from clusters of those three cDNA libraries, with an E-value less than 10E-5 result of the COG BLAST grouped into the general functional class as assigned by COG.

Once constructed, approximately 2300 phage plaques were picked and ultimately sequenced for each of the five cDNA libraries; generating a total of 9601 high quality sequences from the midgut tissue of *L. longipalpis*. These sequences have been submitted to the NCBI EST database under accession numbers EW987149 – EW996682. Table 1 summarizes the results of sequence quality and bioinformatics analysis of each library and the combination of all libraries by the number of sequences analyzed, the number of high quality sequences used in the bioinformatics analysis, the number of contigs, the number of singletons and the average number of sequences per contig. Each library generated a similar number of sequences and sequence recovery from the phage plaques ranged from 79–85%. After discarding low quality sequences, each library retained 71–80% sequences with an average of 73% of the total 11,520 phage producing high quality sequence data. Clustering similar sequences into contigs, based on sequence homology, produced a comparable number of contigs for each library as well as a similar number of singletons. The comparable number of high quality sequences, contigs and singletons produced from each library allows for a better comparison between the sequence abundance of specific molecules of interest and the respective biological condition of the midgut under which they were recovered. The average number of sequences used in the cluster of the contigs varied slightly between libraries. The BF, PBMD, and PBMDi cDNA libraries contained an average sequence per cluster ratio of 8.4, 8.10 and 8.76, respectively. The SF cDNA library had a sequence per cluster ratio of 6.86 and the BFi cDNA library produced an average of 6.34 sequences per cluster. The combining of all cDNA library sequences produced 655 contigs, 2279 singletons and an average of 9.45 sequences per contig. Each cluster was assigned a putative function and placed in a functional class based on the sequence homology to molecules identified by the BLAST results from the NCBI non-redundant protein, the Gene Ontology, the conserved domain, rRNA and mitochondrial databases. Figure 1 shows an overall view of sequence abundance of the functional classes that occurs during the processes of sugar feeding, blood feeding and after the digestion of the blood meal. The clusters of those three cDNA libraries, with an E-value less than 10E-5 result of the KOG BLAST, were grouped according to the general functional class. Although this is a summation of a large number of different clusters, the total number of sequences in each functional class can highlight overall trends that are potentially important in the processes of blood feeding and digestion.

**Table 1:** Overall examination of the 5 individual cDNA libraries and the combined analysis

	**SF**	**BF**	**BFi**	**PBMD**	**PBMDi**	**Combined**
**Sequences analyzed**	1822	1970	1928	1953	1928	9601
**High quality sequences**	1646	1845	1683	1650	1647	8471
**Contigs**	148	137	156	125	117	655
**Singletons**	631	694	694	638	622	2279
**Sequences/contig**	6.86	8.40	6.34	8.10	8.76	9.45

Following is a more detailed description of the most abundant transcripts identified in this analysis:

### Proteases

Proteases were among the most abundant transcripts captured in the random sequencing of the midgut cDNA libraries and included trypsin-like serine proteases, chymotrypsins, carboxypeptidases, and an astacin-like metalloprotease. Table 2 shows the putative proteases identified in the midgut transcriptome. The Sanger Institute's *Lutzomyia longipalpis *EST database was searched using BLAST to find the best matches and results are shown with the corresponding E value. The proteases described here are most similar to those described in the sand fly *Phlebotomus papatasi*, the mosquitoes *Aedes aegypti *or *Anopheles gambie*, with the exception that cluster 91 encodes a putative carboxypeptidase that shares homology with a molecule from the beetle *Tribolium castaneum*. Table 3 shows the transcript producing a full length, high quality sequence for each cluster and the putative function of the identified transcripts. The number of sequences that each cluster contributed to each of the cDNA libraries is also shown and from this it can be seen that most proteases are more abundant, as expected, in the blood fed (BF) and blood fed-Leishmania infected (BFi) libraries. An interesting observation is that cluster 18, which encodes a putative trypsin, is most abundant in the SF, PBMD and PMBDi cDNA libraries, indicating that this putative trypsin may have a role other than blood meal digestion or is produced and stored prior to the ingestion of a blood meal. Table 4 describes the predicted localization, molecular weight and isoelectric point of these proteases. All of the identified proteases posses a potential signal peptide and the molecular weight and isoelectric point given is that of the predicted mature and secreted protein.

**Table 2:** Putative midgut-associated proteases; best matched results and corresponding E values from BLAST inquiries of a GenBank-derived non-redundant protein database and *Lutzomyia longipalpis *EST database

**Cluster**	**Best match to non-redundant protein database**	**NR E value**	**Best match to *Lutzomyia *EST database**	***Lutzomyia *E value**	**GenBank**
35	trypsin 4 [*P. papatasi*]	1.E-101	NSFM-61a01	3.E-140	ABM26904
18	trypsin 1 [*P. papatasi*]	8.E-79	SFM-03g02	3.E-132	ABM26905
83	trypsin 3 [*P. papatasi*]	6.E-94	NSFM-113g08	1.E-125	EU124590
60	trypsin 2 [*P. papatasi*]	4.E-67	NSFM-48a06	2.E-137	EU124582
291	trypsin-eta, putative [*A. aegypti*]	7.E-55	NSFM-15d03	6.E-157	EU124595
33	chymotrypsin [*P. papatasi*]	1.E-96	NSFM-95b07	5.E-143	EU124576
32	chymotrypsin [*P. papatasi*]	8.E-97	NSFM-61f07	1.E-137	EU124575
64	larval chymotrypsin-like protein precursor [*A. aegypti*]	1.E-79	SFM-01b03	1.E-130	EU124583
87	chymotrypsin [*P. papatasi*]	3.E-79	NSFM-96h06	2.E-148	EU124591
30	chymotrypsin [*P. papatasi*]	2.E-94	NSFM-29b07	5.E-133	EU124573
31	chymotrypsin [*P. papatasi*]	5.E-96	NSFM-129f09	1.E-141	EU124574
58/59	ENSANGP00000019623 [*A. gambiae*]	3.E-57	NSFM-121h10	6.E-131	EU124581
104	carboxypeptidase [*A. aegypti*]	1.E-126	SFM-05c11	1.E-221	EU124592
107	carboxypeptidase [*A. aegypti*]	1.E-114	NSFM-146a05	1.E-226	EU124593
91	similar to CG8560-PA [*T. castaneum*]	2.E-82	NSFM-32d09	8.E-199	EU124594

**Table 3:** Putative midgut-associated proteases; putative function and sequence distribution contributed from each cDNA library

			**Number of sequences**					
**Cluster**	**Clone**	**Putative function**	**SF**	**BF**	**BFi**	**PBMD**	**PBMDi**	**Total**
35	LJGFiM23_B07	Trypsin	3	55	34	0	0	92
18	LJGUL-P03_G08	Trypsin	136	6	15	109	168	434
83	LJGFM-P03_E11	Trypsin	8	7	3	7	1	26
60	LJGU-l-5_D05	Trypsin	8	4	8	10	8	38
291	LJGFiM26_A01	Serine protease	2	0	1	0	2	5
33	LJGFM-P04_B01	Chymotrypsin	3	51	22	1	0	77
32	LJGFiL10_C10	Chymotrypsin	0	2	5	0	0	7
64	LJGFM-P03_C01	Chymotrypsin	0	17	17	0	1	35
87	LJGF-l-8_E03	Chymotrypsin	1	14	8	0	2	25
30	LJGDIL5_B09	Chymotrypsin	12	1	3	1	4	21
31	LJGFM-P01_C04	Chymotrypsin	3	4	2	0	0	9
58/59	LJGUL-P01_B07	Astacin-like metalloprotease	28	7	1	1	4	41
104	LJGFL_P01_F01	Carboxypeptidase	0	14	3	0	0	17
107	LJGFL_P03_G11	Carboxypeptidase	6	5	7	0	0	18
91	LJGFiM22_C05	Carboxypeptidase	1	8	8	1	1	19

**Table 4:** Putative midgut-associated proteases; localization, molecular weight and isoelectric point of putative midgut proteins

**Cluster**	**Putative function**	**Gene name**	**Localization**	**Molecular weight (kDa)**	**Isoelectric point**
35	Trypsin	*Lltryp1*	Secreted	26.2	6.32
18	Trypsin	*Lltryp2*	Secreted	26.0	4.95
83	Trypsin	*LuloTryp3*	Secreted	26.0	5.67
60	Trypsin	*LuloTryp4*	Secreted	26.1	5.52
291	Serine protease	*LuloSerPro*	Secreted	29.0	8.26
33	Chymotrypsin	*LuloChym1A*	Secreted	26.8	6.55
32	Chymotrypsin	*LuloChym1B*	Secreted	26.6	6.40
64	Chymotrypsin	*LuloChym2*	Secreted	25.8	6.74
87	Chymotrypsin	*LuloChym3*	Secreted	27.6	4.77
30	Chymotrypsin	*LuloChym4*	Secreted	26.9	5.86
31	Chymotrypsin	*LuloChym5*	Secreted	26.9	6.19
58/59	Astacin-like metalloprotease	*LuloAstacin*	Secreted	28.0	5.04
104	Carboxypeptidase	*LuloCpepA1*	Secreted	45.8	5.36
107	Carboxypeptidase	*LuloCpepA2*	Secreted	46.0	5.41
91	Carboxypeptidase	*LuloCpepB*	Secreted	45.9	4.73

### Trypsin

**Figure 2:**
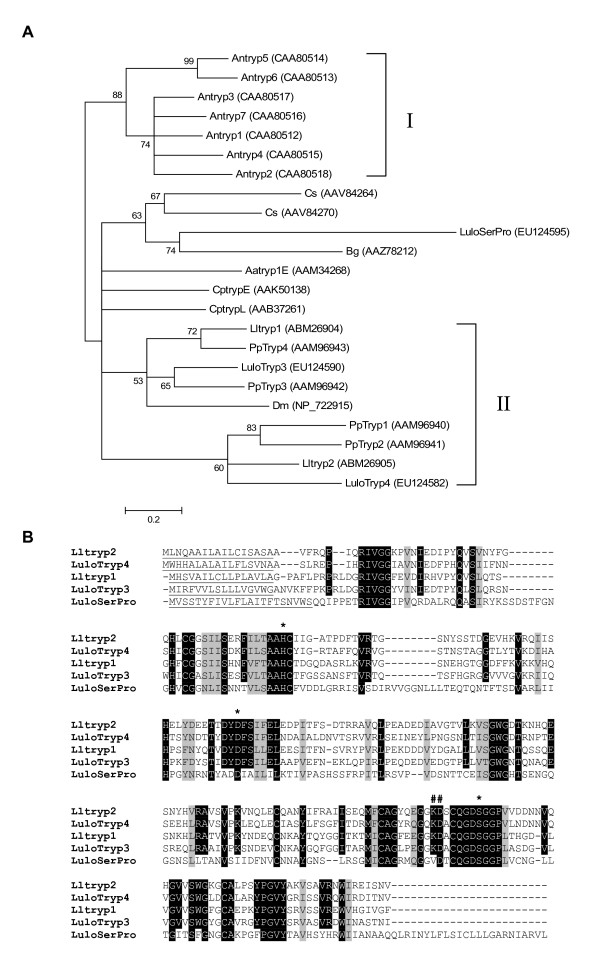
Sequence analysis of trypsin-like serine proteases. (A) Phylogenetic analysis of amino acid sequences from *Anopheles gambiae *(Antryp), *Culicoides sonorensis *(Cs), *Blattella germanica *(Bg), *Lutzomyia longipalpis *(Lulo and Ll), *Phlebotomus paptasi *(Pp), *Aedes aegypti *(Aa), *Drosophila melanogaster *(Dm) and *Culex pipiens quinquefasciatus *(Cp). Node support is indicated by bootstrap values and accession numbers given in parenthesis. (B) Multiple sequence alignment of *Lutzomyia longipalpis *putative trypsin molecules. Predicted secretion signal peptides are underlined, catalytic residues marked by (*) and residues determining substrate specificity marked by (#).

Four trypsin-like transcripts were identified in the transcriptome with high homology to the described *P. papatasi *midgut trypsins [3,6]. Clusters 18, 35, 60 and 83 are similar to *P. papatasi Pptryp1*, *Pptryp4*, *Pptryp2*, and *Pptryp3*, respectively. Recently, two transcripts from *L. longipalpis *midgut EST sequencing were partially characterized and named *Lltryp1*, which corresponds with Cluster 35 identified in our cDNA libraries, and *Lltryp2*, which corresponds with Cluster 18 [3]. *Lltryp2 *was found in highest abundance, 434 sequences, with the unique sequence distribution among the five cDNA libraries in that most sequences were contributed by the sugar fed and post blood meal digestion groups. In order of decreasing abundance of sequences are *Lltryp1*, *LuloTryp4*, and *LuloTryp3*. *LuloTryp3 *and *LuloTryp4 *had relatively homogenous sequence distribution among the cDNA libraries, although *LuloTryp3 *was underrepresented in the PMBDi cDNA library with only one sequence identified. The distribution of *Lltryp1 *sequences between the cDNA libraries correlates with reverse transcriptase-PCR results published showing the expression of *Lltryp1 *during the presence of a blood meal in the female sand fly midgut [3]. Further information about the putative trypsin molecules can be found in Table 4, showing the range of molecular weight ranging from 26.0 to 26.2 kDa. The isoelectric points (pI) of these putative trypsin vary with Lltryp1 having a higher pI of 6.32, Lltryp has a lower pI of 4.95, and LuloTryp3 and LuloTryp4 have similar pI of 5.67 and 5.52, respectively. Phylogenetic analysis of amino acid sequences from Dipteran trypsin molecules and a trypsin from *Blattella germanica *resulted in two major clades, one containing the *A. gambiae *trypsin molecules (group I) and another containing the remaining sequences. Within the other major clade the sand fly trypsins from *L. longipalpis *and *P. papatasi *form two subclades (Group II) (Figure 2A). As previously published[3], Pptryp1 and Pptryp2 form a clade apart from the clade containing Pptryp3 and Pptryp4. The putative trypsin molecules identified in *L. longipalpis *midgut share a high homology with the *P. papatasi *molecules, being grouped into the same clades. Multiple sequence alignment of the trypsin molecules of *L. longipalpis *depicts the potential secretory signal peptide, the H/D/S catalytic site residues and substrate specifying residues (Figure 2B).

A novel midgut-associated serine protease, *LuloSerPro*, was identified in the sequencing and annotation of these midgut cDNA libraries. *LuloSerPro *is predicted to be secreted and have a mature molecular weight of 29.0 kDa, slightly larger than the other trypsin-like serine proteases in the midgut, and has an unusually high predicted pI of 8.26 (Table 4). This molecule, while found in low abundance, was present in the sugar fed, blood fed-Leishmania infected, and post blood meal digestion-Leishmania infected cDNA libraries (Table 3). Phylogenetic analysis and multiple sequence alignments of the midgut trypsin molecules and *LuloSerPro *show that while this molecule is very similar to other trypsin molecules and retains the catalytic residues, this is a distinctly different serine protease (Figure 2). Additionally, there is a difference in the residues that determine the substrate specificity (Lys to Val) between the other midgut trypsins and *LuloSerPro *(Figure 2B).

### Chymotrypsin

Chymotrypsin is another serine protease found in abundance in the midgut of this hematophage midgut. This study identified five clusters with homology to chymotrypsin molecules described in *P. papatasi *and one cluster with homology to a putative larval chymotrypsin found in *A. aegypti *(Tables 2, 3, 4). Clusters 33, 32, 64, 87, 30 and 31 were named *LuloChym1A*, *LuloChym1B*, *LuloChym2*, *LuloChym3*, *LuloChym4 *and *LuloChym5*, respectively. *LuloChym4 *was found in higher abundance in the sugar fed cDNA library and *LuloChym5 *sequences were found in relatively equal numbers between blood fed and sugar fed cDNA libraries. In contrast the other chymotrypsin molecules appear in highest abundance in the blood fed and blood fed-Leishmania infected cDNA libraries (Table 3). According to sequence numbers between the cDNA libraries it appears that chymotrypsin transcription is quiescent after the blood meal has been digested and excreted. The *L. longipalpis *chymotrypsin sequences have a predicted molecular weight of mature and secreted protein ranging from 25.8 to 27.6 kDa (Table 4).

**Figure 3:**
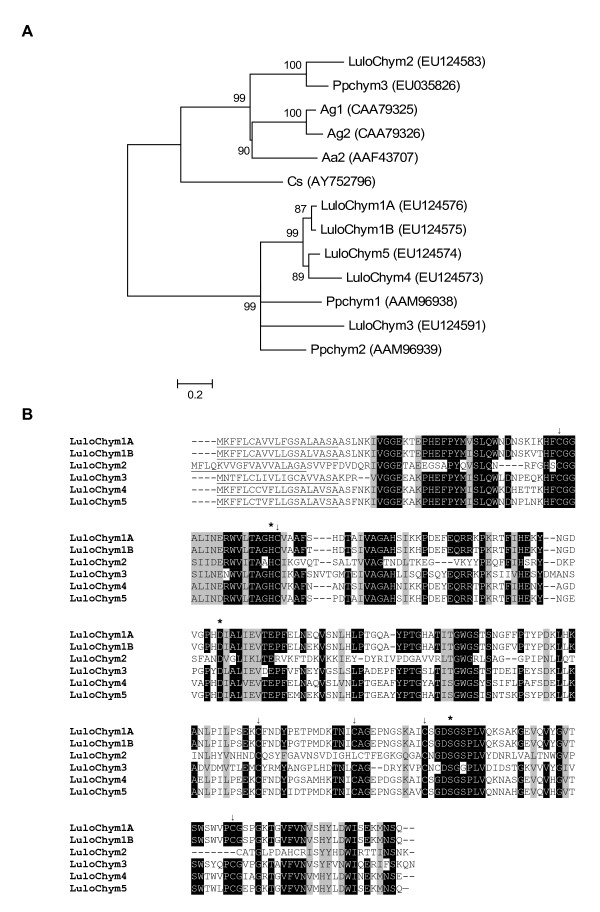
Chymotrysin sequence analysis. (A) Phylogenetic analysis of chymotrypsin sequences from *Phlebotomus papatasi *(Pp), *Lutzomyia longipalpis *(Lulo), *Anopheles gambiae *(Ag), *Aedes aegypti *(Aa), and *Culicoides sonorensis *(Cs). Accession numbers are shown in parenthesis and node support indicated by the bootstrap values. (B) Sequence comparison of midgut putative chymotrypsin molecules. The probably signal peptide is underlined, the catalytic residues inidated by (*) and conserved cysteine residues marked with (↓).

Phylogenetic analysis of chymotrypsin amino acid sequences show that there is conservation in sequence homology between *L. longipalpis *chymotrypsin and *P. papatasi *chymotrypsin molecules (Figure 3A). LuloChym1A, LuloChym1B, LuloChym4 and LuloChym5 form a subclade within a clade containing only sand fly chymotrypsin molecules. The short phylogenetic distance between LuloChym1A and LuloChym1B and the 95% amino acid identity they share suggests that these transcript sequences may represent polymorphisms. Further comparisons between the amino acid sequences of the midgut-associated chymotrypsin molecules show that the cysteine and catalytic residues H/D/S are conserved (Figure 3B).

### Carboxypeptidases

**Figure 4:**
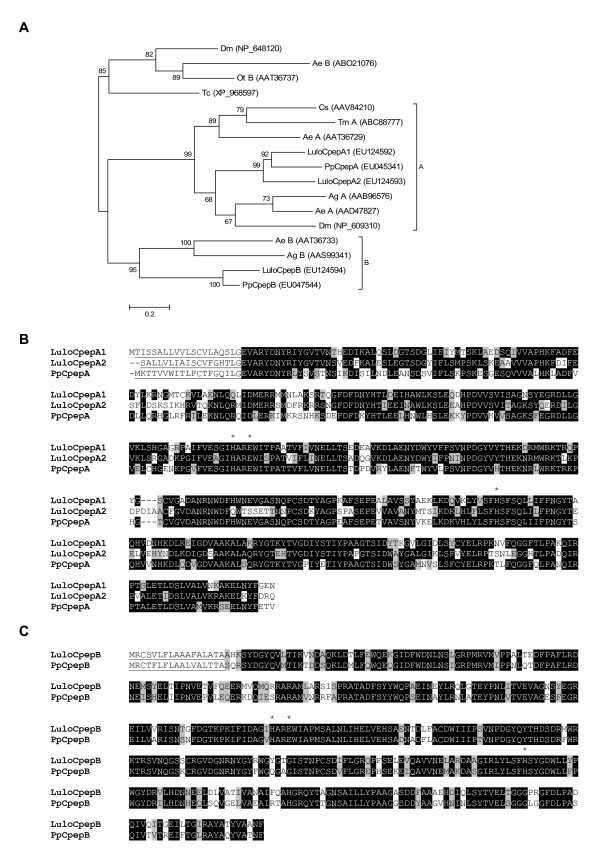
Analysis of putative carboxypeptidase molecules. (A) Phylogenetic analysis of carboxypeptidases from *Lutzomyia longipalpis *(Lulo), *Phlebotomus papatasi *(Pp), *Ochlerotatus triseriatus *(Ot), *Aedes aegypti *(Ae), *Anopheles gambiae *(Ag), *Drosophila melanogaster *(Dm), *Tribolium castaneum *(Tc), *Tenebrio molitor *(Tm) and *Culicoides sonorensis *(Cs). GenBank accession numbers are in parenthesis and node support is indicated by bootstrap values. (B) Sequence alignment of putative carboxypeptidase A molecules identified from the midgut of *Lutzomyia longipalpis *(Lulo) and *Phlebotomus paptasi *(Pp). Predicted catalytic residues are marked with (*). (C) Sequence alignment of putative carboxypeptidase B molecules identified from the midgut of *Lutzomyia longipalpis *(Lulo) and *Phlebotomus paptasi *(Pp). Predicted catalytic residues are marked with (*).

The three longest transcripts encoding putative proteases identified in the analysis are similar to zinc metallocarboxypeptidases found in other insects and significant similarity to ESTs from the Sanger Institute database (Table 2). These transcripts from clusters 104, 107 and 91 were named *LuloCpepA1*,*LuloCpepA2 *and *LuloCpepB*, have molecular weights of 45.8, 46.0 and 45.9 kDa and a pI of 5.36, 5.41 and 4.73, respectively (Table 4). Although *LuloCpepA2 *appears to be an incomplete transcript with a 5' truncation, based on homology and predicted signal peptide sequences a putative mature protein can be used in further characterization and comparison. Most of the sequences grouped to produce the carboxypeptidase clusters were captured from the blood fed library, suggesting that these molecules are likely induced by the ingestion or presence of blood in the midgut of the sand fly (Table 3). The classification of these molecules as members of the A or B class of metallocarboxypeptidases was determined by the output from phylogenetic analysis of the amino acid sequences (Figure 4A). The phylogenetic tree produced by this analysis shows distinct clades containing insect sequences nearly all annotated as either carboxypeptidase A or carboxypeptidase B molecules. The high node support values of the sand fly carboxypeptidases in the phylogenetic tree imply conservation of these molecules when comparing the Old World sand fly *P. papatasi *and that of the New World sand fly *L. longipalpis*. Similarity between the two sand flies, with regards to the carboxypeptidase molecules, can be seen in amino acid sequence alignments, depicting the high level of identity and retention of the catalytic residues necessary for metallocarboxypeptidase activity (Figure 4B, 4C). Furthermore, the amino acid sequence alignment depicts the incongruousness that separates LuloCpepA1 from LuloCpepA2 (Figure 4B).

### Astacin

**Figure 5:**
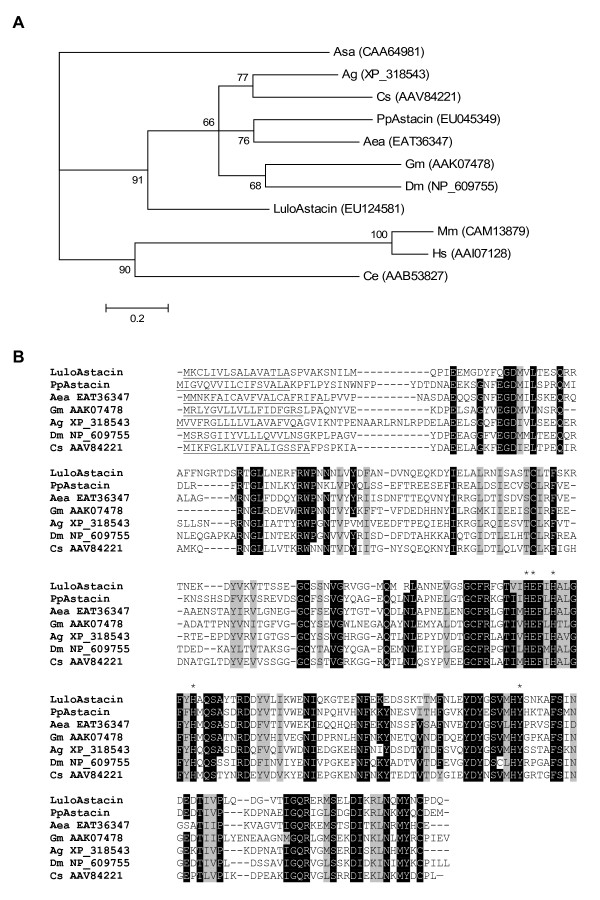
Astacin-like metalloprotease sequence comparison and analysis. (A) Phylogenetic analysis of amino acid sequences from *Lutzomyia longipalpis *(Lulo), *Phlebotomus papatasi *(Pp), *Mus musculus *(Mm), *Homo sapiens *(Hs), *Glossina morsitans morsitans *(Gm), *Drosophila melanogaster *(Dm), *Aedes aegypti *(Aea), *Caenorhabditis elegans *(Ce), *Anopheles gambiae *(Ag), *Astacus astacus *(Asa) and *Culicoides sonorensis *(Cs). Node support is indicated by the bootstrap values. (B) Multiple sequence alignment of Dipteran astacin-like molecules. Predicted signal peptide sequence is underlined and the residues likely necessary for catalytic activity are marked with (*).

A putative zinc metalloprotease was identified as a likely astacin-like molecule based on results for a search of the conserved domains database. This molecule was derived from clusters 58 and 59, both encoding the same putative protein, but separated due to differing lengths of 5'- and 3'- UTRs by the bioinformatics software. The astacin-like metalloprotease was named LuloAstacin and is predicted to have a molecular weight or 28 kDa once secreted and pI of 5.36 (Table 4). LuloAstacin was most abundant in the sugar fed cDNA library in contrast to PpAstacin, an astacin-like molecule identified in *P. papatasi *midgut, which was most abundant in the blood fed cDNA library (Table 3). Phylogenetic analysis of other putative astacin amino acid sequences illustrate that one clade is an assemblage of the Dipteran sequences. LuloAstacin branches out of the subclade containing PpAstacin and away from the other Dipteran sequences (Figure 5A). Further differences in amino acid sequence can be visualized in the multiple sequence alignment of Dipteran astacins and while LuloAstacin diverges from the other astacin molecules, the residues responsible for zinc-binding and activity are conserved (Figure 5B).

### Peritrophin-like proteins

**Figure 6:**
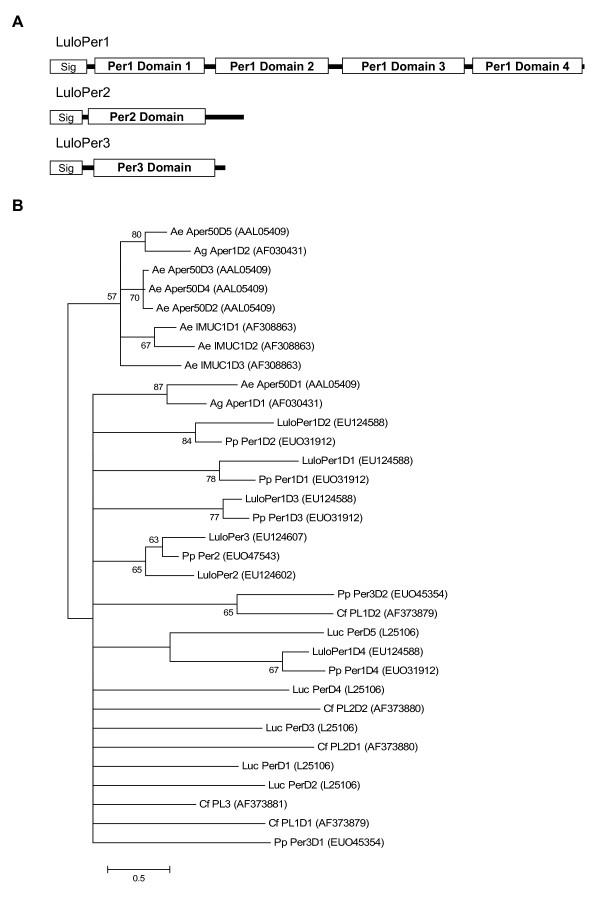
Characterization of peritrophin sequences. (A) Diagrammatic representation of *Lutzomyia longipalpis *peritrophin-like molecules showing the predicted signal peptide and chitin binding domains. (B) Phylogenetic analysis of predicted chitin binding domains of peritrophin molecules from *Aedes aegypti *(Ae), *Anopheles gambiae *(Ag) *Ctenocephalides felis *(Cf), *Lucilia cuprina *(Luc), *Phlebotomus papatasi *(Pp), *Lutzomyia longipalpis *(Lulo). Accession numbers are given in parenthesis and bootstrap values indicate node support.

A number of molecules were identified as containing chitin binding domains based on results from the conserved domains database (Tables 5, 6, 7). Three of the transcripts resembled previously identified peritrophin molecules based on sequence homology with peritrophin-A domains. The most abundant of these putative peritrophin transcripts was named *LuloPer1 *(Cluster 77/78) and was overrepresented in the blood fed *Leishmania*-infected cDNA library and encodes a likely secreted protein of 27.8 kDa (Tables 6 and 7). LuloPer1 consists of four chitin-binding domains (Fig. 6A); contrasting the other two peritrophin molecules, LuloPer2 and LuloPer3, which are molecules of a single chitin-binding domain (Figure 6). *LuloPer2 *and *LuloPer3 *sequences originated in higher numbers from blood fed midgut cDNA libraries and were in relatively equal numbers between the infected and uninfected sand flies. These small putative peritrophins are predicted to have a mature molecular weight of 9.2 and 7.5 kDa and isoelectric points of 4.38 and 3.8 for LuloPer2 and LuloPer3, respectively (Table 7). LuloPer1 is likely to have a role in cross linking chitin fibrils that will form the peritrophic matrix around the ingested blood bolus. LuloPer2 and LuloPer3 may have roles in capping the ends of chitin fibrils or sequestering free chitinous molecules within the midgut lumen. However, the two sequences share only 39% identity and 44% similarity, conserving primarily the cysteine residues, suggesting they may have very different ligand specificities or roles in peritrophic matrix formation or chitin management within the midgut (data not shown). Phylogenetic analysis of the individual chitin-binding domains from several other insect peritrophin and mucin molecules demonstrates conservation of the LuloPer1 domain arrangement when compared with *P. papatasi *PpPer1, suggesting that if the domains are gene duplication events that those events occurred prior to speciation (Figure 6). Additionally, the small putative peritrophin molecules domains from LuloPer2 and LuloPer3 form a clade containing another chitin-binding domain from a small peritophin of *P. papatasi *(Figure 6).

**Table 5:** Putative midgut-associated peritrophin proteins; best matched results and corresponding E values from BLAST inquiries of a GenBank-derived non-redundant protein database and *Lutzomyia longipalpis *EST database

**Cluster**	**Best match to non-redundant protein database**	**NR E value**	**Best match to *Lutzomyia *EST database**	***Lutzomyia *E value**	**GenBank**
77/78	similar to CG7248-PA [*T. castaneum*]	1.E-17	NSFM-114b12	5.E-155	EU124588
114	similar to CG4778-PA [*T. castaneum*]	1.E-12	NSFM-67f02	4.E-06	EU124602
171	ENSANGP00000013237 [*A. gambiae*]	4.E-10	NSFM-67f02	6.E-07	EU124607
274	conserved hypothetical protein [*A. aegypti*]	6.E-67	NSFM-35c11	3.E-28	EU124616

**Table 6:** Putative midgut-associated peritrophin proteins; putative function and sequence distribution contributed from each cDNA library

			**Number of sequences**					
**Cluster**	**Clone**	**Putative function**	**SF**	**BF**	**BFi**	**PBMD**	**PBMDi**	**Total**
77/78	LJGFiM27_H09	Peritrophin	0	6	22	0	0	28
114	LJGUM-P04_G10	Peritrophin	1	7	9	0	0	17
171	LJGFL_P03_H05	Peritrophin	1	4	4	1	0	10
274	LJGFiM24_D03	Chitin binding	1	0	4	1	0	6

**Table 7:** Putative midgut-associated peritrophin proteins; localization, molecular weight and isoelectric point of putative midgut proteins

**Cluster**	**Putative function**	**Gene name**	**Localization**	**Molecular weight (kDa)**	**Isoelectric point**
77/78	Peritrophin	*LuloPer1*	Secreted	27.8	5.00
114	Peritrophin	*LuloPer2*	Secreted	9.2	4.38
171	Peritrophin	*LuloPer3*	Secreted	7.5	3.80
274	Chitin binding	*LuloChiBi*	Secreted	20.9	6.65

In addition to the putative peritrophin molecules a transcript with homology to a predicted chitin-binding domain was identified from the clustering of 6 sequences collected primarily from the blood fed *Leishmania*-infected cDNA library. This domain has homology to a much larger chitin-binding domain than those found in the putative peritrophin molecules and the identified transcript, LuloChiBi, has one of these domains and is predicted to be a mature molecular weight of 20.9 kDa (Table 7).

### Microvillar proteins

Among the most abundant sequences identified in the cDNA libraries were transcripts encoding putative microvillar-associate proteins with homology to insect allergens identified in *Periplaneta americana *and *Blattella germanica *(Table 8). By BLAST analysis high homology was also found to molecules in the mosquito *Aedes aegypti*. In order of decreasing overall sequence abundance, clusters 27, 29, 48, 66 and 36 were named *LuloMVP1*, *LuloMVP2*, *LuloMVP3*, *LuloMVP4 *and *LuloMVP5*, respectively (Table 9). In general the microvillar proteins were most abundant in the blood fed cDNA libraries; although, *LuloMVP3 *(cluster 48) sequences were underrepresented in the blood fed cDNA libraries and was relatively equally identified in the sugar fed and post-blood meal ingestion cDNA. LuloMVP1, LuloMVP2 and LuloMVP5 are of nearly equal mature molecular weight of 21 kDa based on the cleavage of the predicted signal peptide present in all of the microvillar proteins and LuloMVP3 and LuloMVP4 are slightly larger; around 23 kDa. A notable difference in the isoelectric point among the microvillar proteins was observed at a predicted value of 8.84 for LuloMVP3, whereas the other microvillar molecules isoelectric point ranges from 4.46 to 5.12 (Table 10).

**Table 8:** Putative midgut-associated microvillar proteins; best matched results and corresponding E values from BLAST inquiries of a GenBank-derived non-redundant protein database and *Lutzomyia longipalpis *EST database

**Cluster**	**Best match to non-redundant protein database**	**NR E value**	**Best match to *Lutzomyia *EST database**	***Lutzomyia *E value**	**GenBank**
27	conserved hypothetical protein [*A. aegypti*]	2.E-46	NSFM-126e12	2.E-100	EU124571
29	conserved hypothetical protein [*A. aegypti*]	2.E-36	NSFM-19a10	6.E-99	EU124572
48	Cr-PII allergen [*P. americana*]	9.E-22	NSFM-68e08	1.E-101	EU124579
66	conserved hypothetical protein [*A. aegypti*]	4.E-27	NSFM-47h07	1.E-117	EU124584
36	putative protein G12 [*A. aegypti*]	6.E-41	NSFM-154e02	3.E-106	EU124577

**Table 9:** Putative midgut-associated microvillar proteins; putative function and sequence distribution contributed from each cDNA library

			**Number of sequences**					
**Cluster**	**Clone**	**Putative function**	**SF**	**BF**	**BFi**	**PBMD**	**PBMDi**	**Total**
27	LJGFiL9_B01	Microvillar protein	5	109	55	0	0	169
29	LJGFM9_F05	Microvillar protein	3	87	40	0	0	130
48	LJGFS_P01_C07	Microvillar protein	15	6	5	18	18	62
66	LJGFiM27_D08	Microvillar protein	1	24	7	0	0	32
36	LJGFS_P04_B01	Microvillar protein	1	60	27	0	0	88

**Table 10:** Putative midgut-associated microvillar proteins; localization, molecular weight and isoelectric point of putative midgut proteins

**Cluster**	**Putative function**	**Gene name**	**Localization**	**Molecular weight (kDa)**	**Isoelectric point**
27	Microvillar protein	*LuloMVP1*	Secreted	21.6	5.09
29	Microvillar protein	*LuloMVP2*	Secreted	21.5	5.12
48	Microvillar protein	*LuloMVP3*	Secreted	23.1	8.84
66	Microvillar protein	*LuloMVP4*	Secreted	23.6	4.46
36	Microvillar protein	*LuloMVP5*	Secreted	21.7	4.67

**Figure 7:**
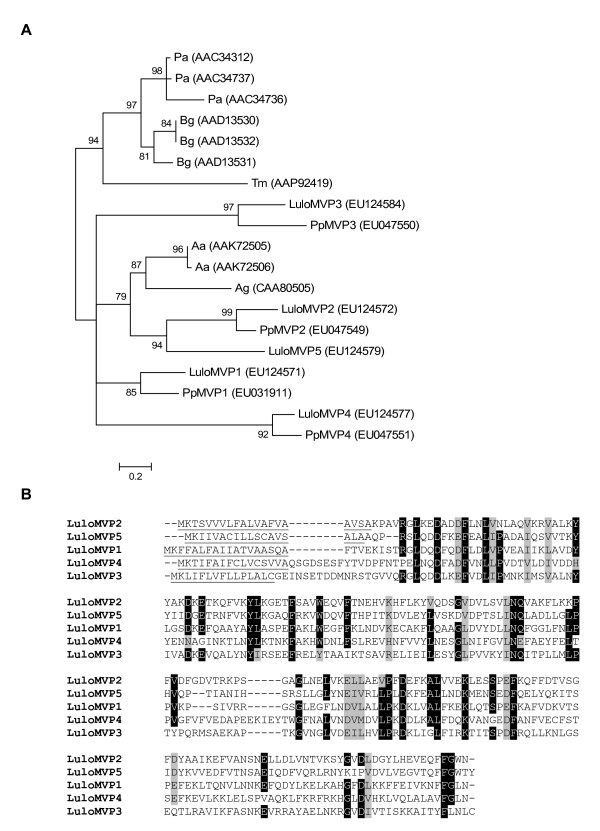
Sequence analysis of microvillar proteins. (A) Phylogenetic analysis of amino acid sequences from *Blattella germanica *(Bg), *Periplaneta americana *(Pa), *Tenebrio molitor *(Tm), *Aedes aegypti *(Aa), *Anopheles gambiae *(Ag), *Phlebotomus papatasi *(Pp) and *Lutzomyia longipalpis *(Lulo). Bootstrap values indicated node support and accession numbers are given in parenthesis. (B) Multiple sequence alignment of the microvillar proteins of *Lutzomyia longipalpis*. The predicted signal secretion peptide is underlined.

The *L. longipalpis *microvillar proteins share respective homology with similar molecules identified in the midgut of *P. papatasi*, as demonstrated by amino acid phylogenetic analysis (Figure 7). The sand fly microvillar proteins are separated from the clade containing cockroaches. Additionally, LuloMVP2 and LuloMVP5 are in a subclade with the microvillar proteins of *A. aegypti *and *A. gambiae *while the other molecules pair with the *P. papatasi *microvillar proteins (Figure 7A). Sequence alignment of the *L. longipalpis *microvillar proteins shows little sequence homology suggesting that the classification of microvillar proteins is rather broad and in fact these molecules may have different functions altogether.

### Oxidative stress molecules

**Figure 8:**
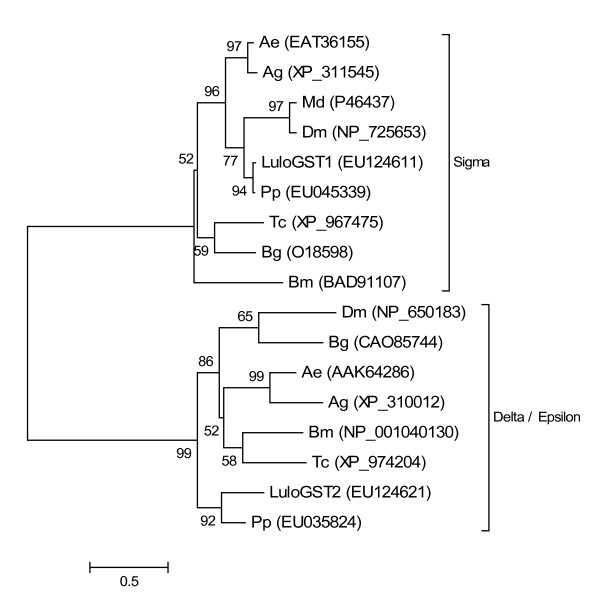
Phylogenetic analysis of glutathione s-transferase molecules. Sequences analysed from *Lutzomyia longipalpis *(Lulo), *Phlebotomus papatasi *(Pp), *Drosophila melanogaster *(Dm), *Aedes aegypti *(Ae), *Anopheles gambiae *(Ag), *Musca domestica *(Md), *Bombyx mori *(Bm), *Tribolium castaneum *(Tc) and *Blattella germanica *(Bg). Accession numbers are given in parentheses and the clades labeled with the respective glutathione s-transferase class.

The sand fly, being an obligate blood feeding insect, must cope with the physiological challenges posed by the digestion of blood which includes the generation of reactive oxygen species (ROS) released by free heme and metabolic radicals produced in abundance during the digestion of the blood meal [7]. Five molecules were identified in the midgut cDNA libraries which have putative roles as antioxidants such as glutathione s-transferase (GST), catalase, copper-zinc superoxide dismutase (SOD) and peroxiredoxin (PRX) (Table 11). In addition to the protection these molecules may impart on the regulation of ROS due to blood meal digestion there is evidence that antioxidants interact with and can impact the outcomes of infection by bacterial and parasitic agents [8]. Two transcripts were identified with homology to GST molecules of the Class Sigma and Class Delta and Epsilon subfamilies and were named *LuloGST1 *and *LuloGST2*, respectively. Phylogenetic analysis of the putative GST molecules supports the separation and classification of into the subfamily classes of Sigma and Delta/Epsilon. Additionally, LuloGST1 is grouped in a subclade with other dipertan GST molecules while LuloGST2 diverges from the dipteran Delta/Epsilon GST molecules (Figure 8). The *LuloGST1 *cluster was generated from sequences from each of the cDNA libraries made and analyzed while *LuloGST2 *consists of one sequence from the sugar fed library and two sequences from the blood fed *Leishmania*-infected cDNA library. Additional antioxidant molecules include a catalase (*LuloCAT*), copper-zinc superoxide dismutase (*LuloSOD*), and peroxiredoxin (*LuloPRX*) of which LuloSOD and LuloPRX are both predicted to be secreted based on the presence of a likely signal peptide sequence. ROS and reactive nitrogen oxide species (RNOS) are important in host defenses against microorganisms and LuloCAT, LuloSOD and LuloPRX are molecules which may serve to regulate and prevent damage of the sand fly midgut by the ROS and RNOS defenses similar to the protective effect of a peroxiredoxin in *Anopheles stephensi *[9].

**Table 11:** Putative midgut-associated oxidative stress molecules; best matched results and corresponding E values from BLAST inquiries of a GenBank-derived non-redundant protein database and *Lutzomyia longipalpis *EST database

**Cluster**	**Best match to non-redundant protein database**	**NR E value**	**Best match to *Lutzomyia *EST database**	***Lutzomyia *E value**	**GenBank**
221	glutathione s-transferase [*A. aegypti*]	8.E-89	NSFM-105e10	9.E-105	EU124611
419	glutathione s-transferase [*A. aegypti*]	2.E-50	NSFM-95g05	2.E-116	EU124621
76	GA13179-PA [*D. pseudoobscura*]	1.E-43	NSFM-144g07	6.E-103	EU124587
79	ferritin heavy chain-like [*G. morsitans*]	2.E-66	NSFM-146d09	7.E-111	EU124589
781	catalase [*A. aegypti*]	1.E-119	NSFM-142e04	3.E-286	EU124624
1709	ENSANGP00000015824 [*A. gambiae*]	1.E-50	NSFM-39d09	1.E-91	EU124625
2557	peroxiredoxins, prx-1, prx-2, prx-3 [*A. aegypti*]	1.E-105	NSFM-34h03	3.E-126	EU124629

Upon the ingestion of a blood meal by a hematophagous insect a large amount of iron and heme is released during digestion. To combat the toxic effects of free iron and the generation of damaging reactive oxygen species ferritin is produced to sequester the iron and hemoglobin that is liberated by the digestion of red blood cells. Ferritin molecules are commonly associated with iron metabolism and it is likely that the molecules identified in this transcriptome engage in metabolic function; however, given the relative size of the blood meal in comparison with the sand fly ferritin molecules within the midgut likely serve a large role in preventing the generation of oxygen radicals by the Fenton reaction. Two transcripts from clusters 76 and 79 were identified with homology to ferritin light-chain and ferritin heavy-chain molecules and were named *LuloFLC *and *LuloFHC*, respectively (Tables 11 and 13). The expression of *LuloFLC *and *LuloFHC *appears to be constitutive based on the number of sequences generated in each cDNA library spanning the condition of sugar fed, blood fed, and post blood meal digestion (Table 12).

**Table 12:** Putative midgut-associated oxidative stress molecules; putative function and sequence distribution contributed from each cDNA library

			**Number of sequences**					
**Cluster**	**Clone**	**Putative function**	**SF**	**BF**	**BFi**	**PBMD**	**PBMDi**	**Total**
221	LJGFM9_A02	Glutathione s-transferase	1	2	1	2	1	7
419	LJGFIL9_G06	Glutathione s-transferase	1	0	2	0	0	3
76	LJGFL_P04_E07	Ferritin light-chain	5	7	8	3	5	28
79	LJGFiM24_C11	Ferritin heavy-chain	3	6	7	4	4	24
781	LJGFiM21_C02	Catalase	0	0	1	0	1	2
1709	LJGD-L10_H10	Cu/Zn superoxide dismutase	0	0	0	1	0	1
2557	LJGDiM21_G01	Peroxiredoxin	0	0	0	0	1	1

**Table 13:** Putative midgut-associated oxidative stress molecules; localization, molecular weight and isoelectric point of putative midgut proteins

**Cluster**	**Putative function**	**Gene name**	**Localization**	**Molecular weight (kDa)**	**Isoelectric point**
221	Glutathione s-transferase	*LuloGST1*	Intracellular	23.3	5.00
419	Glutathione s-transferase	*LuloGST2*	Intracellular	24.8	6.41
76	Ferritin light-chain	*LuloFLC*	Secreted	24.4	6.68
79	Ferritin heavy-chain	*LuloFHC*	Secreted	21.9	4.92
781	Catalase	*LuloCat*	Intracellular	57.7	8.11
1709	Cu/Zn superoxide dismutase	*LuloSOD*	Secreted	19.8	5.63
2557	Peroxiredoxin	*LuloPRX*	Secreted	25.0	6.66

### Serine protease inhibitors

Two types of serine protease inhibitors were identified in the cDNA libraries; a single sequence with homology to SERPIN and a cluster of 17 sequences with homology to a Kazal-type serine protease inhibitor (Tables 14, 15, 16). SERPIN molecules within the midgut of the sand fly may serve to counteract damaging proteases produced by microorganisms; however LuloSRPN lacks a predicted signal peptide sequence and thus may serve an intracellular housekeeping function. *LuloKZL*, identified from cluster 112, is a small molecule of 6.3 kDa and is predicted to be secreted. Comparison of LuloKZL with Kazal-type serine protease inhibitors found in a transcriptome analysis of the midgut of *P. papatasi *identified PpKZL1 as a highly conserved homolog (data not shown). Kazal-type protease inhibitors, such as rhodniin and infestin identified in *Rhodnius prolixus *and *Triatoma infestans*, respectively, have been characterized as thrombin inhibitors; thereby these molecules would prevent coagulation of ingested blood to facilitate successful digestion of the blood meal [10,11]. *LuloKZL *sequences are more abundant prior to and during blood meal digestion based on the number of sequences in the sugar fed, blood fed and post blood meal digestion cDNA libraries. Additionally, *LuloKZL *was not identified in an EST analysis of whole sand fly *L. longipalpis *and is therefore more likely a midgut-specific molecule found in abundance only in the alimentary tissue [5]. Thus, a prudent hypothesis would be that LuloKZL serves a similar function, allowing the blood bolus to remain in a colloidal suspension within the gut to facilitate peristalsis and digestion.

**Table 14:** House keeping and low abundant transcripts from the midgut of *L. longipalpis*; best matched results and corresponding E values from BLAST inquiries of a GenBank-derived non-redundant protein database and *Lutzomyia longipalpis *EST database

**Cluster**	**Best match to non-redundant protein database**	**NR E value**	**Best match to *Lutzomyia *EST database**	**E value**	**GenBank**
128	GAPDH II [*D. pseudoobscura*]	1.E-168	SFM-03d02	2.E-161	EU124605
195	fructose-bisphosphate aldolase [*A. aegypti*]	1.E-138	NSFM-99b02	5.E-153	EU124609
189	sugar transporter [*A. aegypti*]	0.E+00	NSFM-46e10	1.E-255	EU124608
200	ENSANGP00000018531 [*A. gambiae*]	0.E+00	SFM-05b09	4.E-225	EU124610
292	cytochrome c oxidase subunit iv [*A. aegypti*]	3.E-58	NSFM-43a12	6.E-97	EU124618
97	ADP/ATP translocase [*L. cuprina*]	1.E-154	NSFM-64b06	2.E-161	EU124598
69	Vacuolar ATP synthase 16 kDa proteolipid subu [*A. aegypti*]	2.E-77	NSFM-95b05	6.E-70	EU124586
67/192	Actin 87E CG18290-PA, isoform A [*D. melanogaster*]	0.E+00	NSFM-41f08	5.E-202	EU124585
112	GA16408-PA [*D. pseudoobscura*]	4.E-10			EU124601
2287	serine protease inhibitor 4 [*A. aegypti*]	3.E-27	NSFM-73e11	3.E-185	EU124627
358	RAS, putative [*A. aegypti*]	1.E-90	NSFM-155h05	2.E-93	EU124620
2556	ENSANGP00000016718 [*A. gambiae*]	1.E-103	NSFM-83c08	4.E-99	EU124628
500	conserved hypothetical protein [A. aegypti]	7.E-08	NSFM-154d08	3.E-09	EU124623
235	peptidoglycan recognition protein LB [*G. morsitans*]	8.E-69	NSFM-81b08	4.E-109	EU124614
1960	defensin isoform B1 [*A. aegypti*]	2.E-12			EU124626
269	40S ribosomal protein S7 ribosomal protein [*C. pipiens*]	3.E-89	NSFM-15f05	1.E-97	EU124615
423	ribosomal protein S20 [*B. mori*]	7.E-56	NSFM-41g09	2.E-59	EU124622
226	ribosomal protein S8 [*A. albopictus*]	6.E-93	NSFM-01c05	9.E-97	EU124612
125	LD16326p [*D. melanogaster*]	1.E-100	NSFM-52a05	2.E-84	EU124604
304	Ribosomal protein L32 CG7939-PC, isoform C [*D. melanogaster*]	1.E-67			EU124619
101	GA20389-PA [*D. pseudoobscura*]	1.E-153	SFM-03h12	2.E-133	EU124599
108	60S acidic ribosomal protein P1 [*S. frugiperda*]	3.E-48	NSFM-163b12	5.E-27	EU124600
119	similar to Drosophila melanogaster CG2099 [*D. yakuba*]	2.E-54	NSFM-100a07	6.E-62	EU124603
40	similar to Neurospecific receptor kinase CG4007-PA [*A. mellifera*]	8.E-01	NSFM-57e04	6.E-140	EU124578
54/55	14.5 kDa salivary protein [*P. duboscqi*]	8.E-41			EU124580
88	bS11M [*A. aegypti*]	2.E-03	NSFM-149f10	1.E-58	EU124596
151	CG14401-PA [*D. melanogaster*]	3.E-06	NSFM-114e07	1.E-11	EU124606
230	conserved hypothetical protein [*A. aegypti*]	8.E-22			EU124613
90	Hypothetical protein C30H6.11[*C. elegans*]	2.E-10	NSFM-55h01	7.E-64	EU124597
276	CG32644-PB [*D. melanogaster*]	2.E-11	NSFM-23d08	8.E-27	EU124617

**Table 15:** House keeping and low abundant transcripts from the midgut of *L. longipalpis*; putative function and sequence distribution contributed from each cDNA library

			**Number of sequences**					
**Cluster**	**Clone**	**Putative function**	**SF**	**BF**	**BFi**	**PBMD**	**PBMDi**	**Total**
128	LJGDiM22_F06	Glyceraldehyde-3-phosphate dehydrogenase	1	1	0	4	7	13
195	LJGDIL8_D01	Fructose-bisphosphate aldolase	0	0	0	3	3	6
189	LJGFL_P04_A09	Sugar transporter	2	3	0	2	1	8
200	LJGD-L2_G11	Enolase	0	1	0	3	2	6
292	LJGFiM27_F04	Cytochrome c oxidase IV	1	2	1	0	1	5
97	LJGDiM25_C06	ADP/ATP translocase	4	0	2	3	6	15
69	LJGDIL9_F01	V-ATPase C-subunit	2	6	4	7	7	26
67/192	LJGFL_P01_G02	Actin	10	16	4	6	2	38
112	LJGUM-P03_G07	Kazal-type serine protease inhibitor	6	4	5	2	0	17
2287	LJGFiM21_F02	Serine protease inhibitor 4	0	0	1	0	0	1
358	LJGFM5_B11	Ras	1	1	2	0	0	4
2556	LJGDiM21_F11	Aquaporin	0	0	0	0	1	1
500	LJGFiL8_B01	Galectin	0	0	1	2	0	3
235	LJGFS_P02_C04	Peptidoglycan recognition protein	3	2	0	0	1	6
1960	LJGDM27_A10	Defensin	0	0	0	1	0	1
269	LJGUM-P03_F07	40S ribosomal protein S7	1	1	1	2	1	6
423	LJGDM25_A04	40S ribosomal protein S20	1	1	0	1	0	3
226	LJGDiM26_A12	40S ribosomal protein S8	1	2	1	2	1	7
125	LJGFiM25_D10	60s ribosomal protein L19	5	2	2	2	2	13
304	LJGF-L-8_E06	60S ribosomal protein L32	1	2	0	0	2	5
101	LJGFiL3_D04	60S acidic ribosomal protein P0	3	0	4	5	5	17
108	LJGU-m-5_A09	60S acidic ribosomal protein P1	6	0	2	6	4	18
119	LJGUS_P03_A12	60S Ribosomal protein L35Ae	10	1	1	3	0	15
40	LJGFiL7_D12	Unknown	6	4	13	25	22	70
54/55	LJGF-l-10_A05	Unknown	7	13	13	11	4	48
88	LJGU-l-10_D11	Unknown	5	2	2	5	2	16
151	LJGFiL1_H05	Unknown	0	2	6	1	2	11
230	LJGFiM22_H04	Unknown	0	1	3	1	2	7
90	LJGU-l-7_C03	Unknown	5	5	5	4	5	24
276	LJGFiL2_F05	Unknown	0	1	2	3	0	6

**Table 16:** House keeping and low abundant transcripts from the midgut of *L. longipalpis*; localization, molecular weight and isoelectric point of putative midgut proteins

**Cluster**	**Putative function**	**Gene name**	**Localization**	**Molecular weight (kDa)**	**Isoelectric point**
128	Glyceraldehyde-3-phosphate dehydrogenase		Intracellular	35.2	7.84
195	Fructose-bisphosphate aldolase		Intracellular	30.8	6.73
189	Sugar transporter		Transmembrane	53.7	7.03
200	Enolase		Intracellular	46.7	6.5
292	Cytochrome c oxidase IV		Intracellular	20.7	9.26
97	ADP/ATP translocase		Transmembrane	33.3	9.87
69	V-ATPase C-subunit		Transmembrane	16.0	8.41
67/192	Actin		Intracellular	41.8	5.29
112	Kazal-type serine protease inhibitor	*LuloKZL*	Secreted	6.3	4.83
2287	Serine protease inhibitor 4	*LuloSRPN*	Intracellular	42.1	4.95
358	Ras		Intracellular	20.5	5.20
2556	Aquaporin		Transmembrane	27.7	8.71
500	Galectin	*LuloGalec*	Intracellular	17.2	7.33
235	Peptidoglycan recognition protein	*LuloPGRP*	Intracellular	21.9	6.75
1960	Defensin	*LuloDEF*	Secreted	7.2	6.89
269	40S ribosomal protein S7		Intracellular	21.9	9.82
423	40S ribosomal protein S20		Intracellular	13.4	10.44
226	40S ribosomal protein S8		Intracellular	23.6	10.72
125	60s ribosomal protein L19		Intracellular	24.0	11.13
304	60S ribosomal protein L32		Intracellular	16.0	11.77
101	60S acidic ribosomal protein P0		Intracellular	34.2	6.23
108	60S acidic ribosomal protein P1		Intracellular	11.5	4.08
119	60S Ribosomal protein L35Ae		Intracellular	16.8	11.24
40	Unknown		Secreted	29.2	9.59
54/55	Unknown		Secreted	14.3	9.13
88	Unknown		Secreted	14.5	7.78
151	Unknown		Secreted	11.9	4.72
230	Unknown		Secreted	11.6	9.95
90	Unknown		Secreted	19.0	3.8
276	Unknown		Secreted	16.6	3.41

### Anti-bacterial molecules

**Figure 9:**
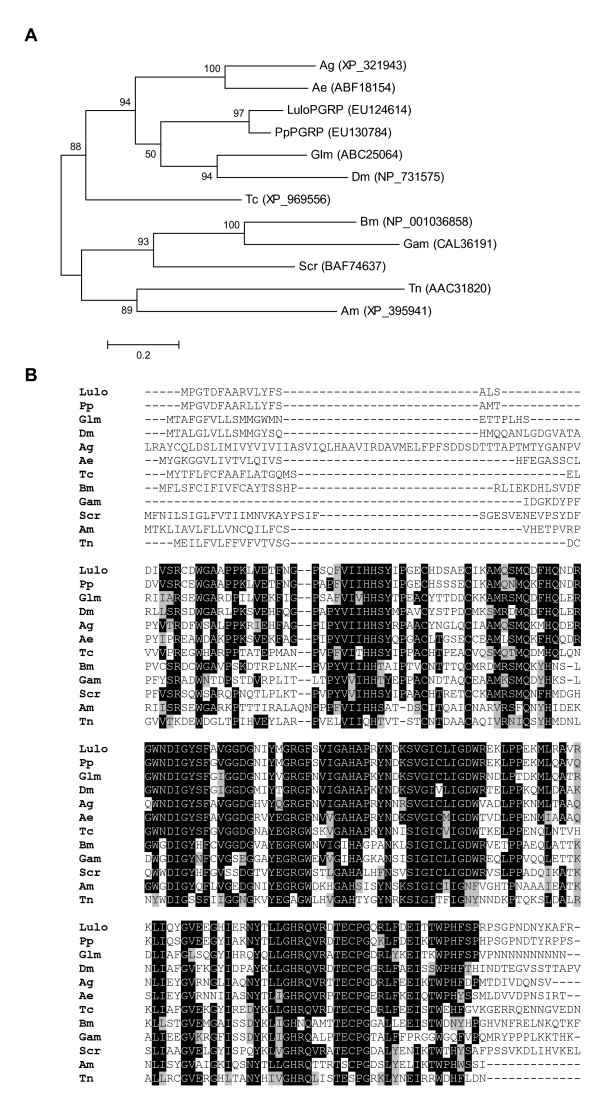
Sequence analysis of peptidoglycan recognition proteins. (A) Phylogenetic analysis of amino acid sequences of peptidoglycan recognition proteins from *Lutzomyia longipalpis *(Lulo), *Phlebotomus papatasi *(Pp), *Anopheles gambiae *(Ag), *Aedes aegypti *(Ae), *Glossina moristans moristans *(Glm), *Drosophila melanogaster *(Dm), *Tribolium castaneum *(Tc), *Apis mellifera *(Am), *Bombyx mori *(Bm), *Galleria mellonella *(Gam), *Trichoplusia ni *(Tn) and *Samia cynthia ricini *(Scr). Accession numbers are in parentheses and bootstrap values indicate node support. (B) Multiple sequence alignment of peptidoglycan recognition proteins. Identical amino acid residues are highlighted black and similar residues are highlighted grey.

Two molecules, originating from clusters 235 and 1960, encode a putative peptidoglycan recognition protein (*LuloPGRP*) and defensin (*LuloDEF*), respectively. LuloPGRP is similar to other predicted peptidoglycan recognition proteins found in *Glossina morsitans morsitans *and mosquitoes and is phylogenetically distinct from lepidopteran molecues (Figure 9). This is the first report of a putative PGRP identified in sand flies and in searching a midgut transcriptome database of *P. papatasi *a molecule was identified with 87% identity. LuloPGRP may serve as a pattern recognition protein, specifically for the conserved structure of peptidoglycan indicated by the conservation of the amino acid sequence among insects, as a component of the sand fly immune system defense against bacterial pathogens (Figure 9). PGRP molecules characterized in *Bombyx mori *and *Trichoplusia ni *have been shown to be expressed primarily in the fat body and hemocytes and it is conceivable that the identification of *LuloPGRP *transcripts arose due to a contamination of the tissue sample [12,13]. It is possible that the midgut tissue of sand flies express a PGRP for protection against microorganisms ingested during sugar and blood feeding as a PGRP was identified as preferentially expressed in the midgut of *Samia cynthia ricini *[14].

**Figure 10:**
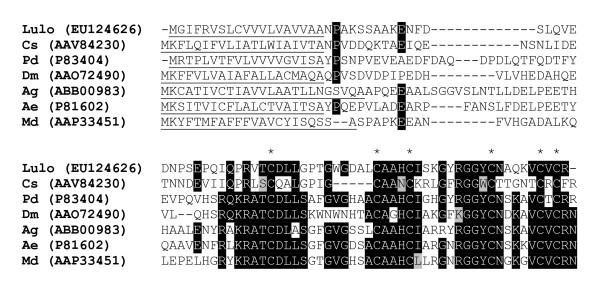
Multiple sequence alignment of putative defensin sequences. Aligned sequences from *Lutzomyia longipalpis *(Lulo), *Culicoides sonorensis *(Cs), *Phlebotomus duboscqi *(Pd), *Drosophila melanogastor *(Dm), *Anopheles gambiae *(Ag), *Aedes aegypti *(Ae) and *Muscus domestica *(Md). Conserved cysteine residues are marked with (*).

Defensins are another type of innate immune defense that insect possesses to ward off pathogenic bacteria. A single sequence, named *LuloDEF*, was identified in the post blood meal digestion midgut cDNA library with homology to a defensin molecule characterized in *A. aegypti*. Like other insect defensin molecules, LuloDEF has a predicted secretion signal peptide and most homology is given by the carboxyl half of the sequence and conservation of cysteine residues (Figure 10). LuloDEF shares 47% identity and 61% similarity with a defensin characterized in *Phlebotomus duboscqi *which is induced by the presence of wild type *Leishmania major *[15]. Both immunity-associated genes, *LuloPGRP *and *LuloDEF*, may have an impact on the progression and result of a midgut infection by Leishmania parasites, either directly or by indirect effects if co-colonization of the midgut with bacteria is an intermediary confounding factor.

### Transcripts differentially expressed by blood feeding and digestion

A comparison between the sugar fed and blood and between the blood fed and post blood meal digestion libraries was conducted using Pearson's chi-square equation to identify overrepresented transcripts within each cluster. As was previously seen in *P. papatasi *a number of digestion-associated transcripts were overabundant in the blood fed cDNA library [6]. We envisioned similar results in the analysis of the *L. longipalpis *midgut cDNA libraries with the enhanced advantage of a cDNA library produced from midguts that had fully processed and excreted the blood meal byproducts. It was our hypothesis that the post blood meal midgut transcript abundance is most similar to the sugar fed midgut transcript abundance prior to a blood meal. Overall, the number of sequences per cluster was similar in the sugar fed cDNA library to those in the post blood meal digestion cDNA library and most transcripts are overrepresented in the blood fed library (Table 17). Several exceptions to both overall observations do occur, however. Most of the microvillar protein transcripts are abundant in the blood fed cDNA library except for *LuloMVP3*, which is highly represented in the sugar fed and post blood meal digestion cDNA libraries. This reinforces the suggestion that the microvillar proteins are likely functionally different molecules grouped solely on homology to previously annotated sequences. In general, proteases appear to be induced by the act of blood feeding or the presence of a blood meal within the midgut; with the exception of *Lltryp2 *which is significantly more abundant in the sugar-fed and also in the post blood meal digestion cDNA libraries and also *LuloAstacin *which is more abundant in the sugar-fed cDNA library (Tables 17 and 18). These molecules may be produced and stored prior to blood feeding for immediate use in digestion or perhaps have a role other than digestion altogether, such as immunity. Other proteases such as *LuloChym4 *and *LuloCpepA2 *are present in higher or near equal numbers in the sugar fed library when compared with that of the blood fed library. Other molecules such as Peritrophin *LuloPer1 *and *LuloPer2*, are also more plentiful in the blood fed cDNA library, suggesting that these molecules may be transcribed only in response to blood feeding. A transcript encoding a predicted protein of unknown function derived from cluster 40 was identified as being most abundant in the post blood meal digestion cDNA library, signifying it may play a role outside of blood meal digestion, such as oogenesis.

**Table 17:** Sequence distribution altered during sugar feeding and blood meal digestion; clusters overrepresented in the sugar-fed, blood-fed and post blood meal digestion midgut cDNA libraries as determined by X^2 ^statistical analysis

**Putative function**	**Cluster #**	**SG**	**BF**	**PBMD**	**P value**	**GenBank**
Microvillar protein (*LuloMVP1*)	27	5	109	0	6.8E-03	EU124571
Microvillar protein (*LuloMVP2*)	29	3	87	0	3.2E-06	EU124572
Microvillar protein (*LuloMVP3*)	48	15	6	18	4.2E-02	EU124579
Microvillar protein (*LuloMVP4*)	66	1	24	0	3.5E-02	EU124584
Microvillar protein (*LuloMVP5*)	36	1	60	0	3.2E-04	EU124577
Trypsin (*Lltryp1*)	35	3	55	0	4.9E-08	ABM26904
Trypsin (*Lltryp2*)	18	136	6	109	4.2E-02	ABM26905
Chymotrypsin (*LuloChym1A*)	33	3	51	1	1.1E-17	EU124576
Chymotrypsin (*LuloChym2*)	64	0	17	0	2.4E-02	EU124583
Astacin-like metalloprotease (*LuloAstacin*)	58	23	5	0	3.6E-16	EU124581
Unknown	40	6	4	25	3.4E-02	EU124578

**Table 18:** Sequence distribution altered during sugar feeding and blood meal digestion; clusters that appear overabundant in the sugar-fed, blood-fed and post blood meal digestion midgut cDNA libraries

**Putative function**	**Cluster #**	**SG**	**BF**	**PBMD**	**P value**	**GenBank**
Peritrophin (*LuloPer1*)	77	0	6	0	4.3E-02	EU124588
Peritrophin (*LuloPer2*)	114	1	7	0	2.0E-05	EU124602
Chymotrypsin (*Lulochym3*)	87	1	14	0	4.2E-02	EU124591
Chymotrypsin (*LuloChym4*)	30	12	1	1	1.7E-03	EU124573
Carboxypeptidase (*LuloCpepA1*)	104	0	14	0	1.4E-02	EU124592
Carboxypeptidase (*LuloCpepA2*)	107	6	5	0	3.40E-02	EU124593
Carboxypeptidase (*LuloCpepB*)	91	1	8	1	2.4E-07	EU124594
60S acidic ribosomal protein P0	101	3	0	5	1.80E-02	EU124599
60S acidic ribosomal protein P1	108	6	0	6	9.2E-05	EU124600
60S Ribosomal protein L35Ae	119	10	1	3	4.2E-02	EU124603

### Transcripts differentially expressed by the presence of Leishmania infantum chagasi

To evaluate the effects of the presence of *L. infantum chagasi *parasites on the transcript abundance in the midgut tissue of the sand fly we compared the number of sequences in each cluster between the blood fed and blood fed Leishmania-infected cDNA library and the post blood meal digestion and post blood meal digestion Leishmania-infected cDNA library using chi-square analysis (Tables 19, 20, 21, 22). We hypothesized that the effects of the parasites presence in the blood engorged sand fly would likely mirror what we had observed in a similar comparison of *P. papatasi *infected with *L. major*. Additionally, we hypothesized that the analysis of the post blood meal digestion midgut tissue would reveal a large number of differentially abundant transcripts as during this time period Leishmania parasites are interacting with the midgut epithelium, replicating, and differentiating to the metacyclic form. In accordance with what we observed previously in blood engorged *P. papatasi *infected with *L. major*, there was an under representation of the microvillar protein transcripts [6]. Similar trends in abundance between infected *P. papatasi *and infected *L. longipalpis *also occur for transcripts encoding the putative digestion enzymes trypsin (*Lltryp2*) and chymotrypsin (*LuloChym1A*). Two other digestive proteases, *LuloAstacin *and *LuloCpepA1*, were identified as differentially abundant in the presence of *L. infantum chagasi *with a reduction in the number of transcripts captured in the blood fed Leishmania-infected library, however only the *LuloCpepA1 *difference was statistically significant. There is a striking contradiction of the modulated abundance of peritrophin transcripts. In the midgut of infected *P. papatasi *peritrophin transcripts decrease whereas in *L. longipalpis *infected with *L. infantum chagasi *has a significant over representation of peritrophin (*LuloPer1*) and over representation of the putative chitin-binding molecule (*LuloChiBi*). There appears to be a downregulation of actin transcripts by the presence of the *L. infantum chagasi *parasites in the midgut. We speculate that this could be a tactic of the parasite to decrease the cytoskeletal rearrangement that occurs after blood feeding as a means of decreasing peristalsis, which may aid in the retention of the parasite within the gut of the sand fly.

**Table 19:** Sequence distribution altered by *Leishmania infantum chagasi*; clusters overrepresented in the blood-fed and blood-fed *Leishmania infantum chagasi*-infected midgut cDNA libraries as determined by X^2 ^statistical analysis

**Putative function**	**Cluster #**	**BF**	**BFi**	**P value**	**GenBank**
Microvillar protein (*LuloMVP1*)	27	109	55	2.0E-04	EU124571
Microvillar protein (*LuloMVP2*)	29	87	40	2.0E-04	EU124572
Microvillar protein (*LuloMVP4*)	66	24	7	4.9E-03	EU124584
Microvillar protein (*LuloMVP5*)	36	60	27	1.6E-03	EU124577
Peritrophin (*LuloPer1*)	77/78	6	22	1.0E-03	EU124588
Trypsin (*Lltryp2*)	18	6	15	2.9E-02	ABM26905
Chymotrypsin (*LuloChym1A*)	33	51	22	2.4E-03	EU124576
Carboxypeptidase (*LuloCpepA1*)	104	14	3	1.3E-02	EU124592
Actin	67/192	16	4	1.3E-02	EU124585
Unknown	40	4	13	1.7E-02	EU124578

**Table 20:** Sequence distribution altered by *Leishmania infantum chagasi*; clusters overrepresented in the post blood meal digestion and post blood meal digestion *Leishmania infantum chagasi*-infected midgut cDNA libraries as determined by X^2 ^statistical analysis

**Putative function**	**Cluster #**	**PBMD**	**PBMDi**	**P value**	**GenBank**
Trypsin (*Lltryp2*)	18	109	168	2.0E-04	ABM26905

**Table 21:** Sequence distribution altered by *Leishmania infantum chagasi*; clusters that appear overabundant in the blood-fed or blood-fed *Leishmania infantum chagasi*-infected midgut cDNA libraries

**Putative function**	**Cluster #**	**BF**	**BFi**	**GenBank**
Chitin binding (*LuloChiBi*)	274	0	4	EU124616
Astacin-like metalloprotease (*LuloAstacin*)	58/59	7	1	EU124581

**Table 22:** Sequence distribution altered by *Leishmania infantum chagasi*; LuloTryp3 appears underrepresented in the post blood meal digestion *Leishmania infantum chagasi*-infected midgut cDNA library

**Putative function**	**Cluster #**	**PBMD**	**PBMDi**	**GenBank**
Trypsin (*LuloTryp3*)	83	7	1	EU124590

In the context of abundant transcripts, the post blood meal digestion midgut infected with *L. infantum chagasi *is relatively quiescent. Only one transcript, encoding a putative trypsin molecule, was identified as significantly different in abundance. *Lltryp2 *sequences were 1.54 times more abundant in the *L. infantum chagasi*-infected post blood meal digestion cDNA library which corroborates the observed overrepresentation of *Lltryp2 *sequences in the blood fed infected cDNA library. It is possible that the increase in sand fly *Lltryp2 *occurs due to the presence of a perceived pathogen or as a consequence of a non-specific perception of contents within the midgut. Conversely, *LuloTryp3 *transcripts were captured at a lower frequency in the *L. infantum chagasi*-infected midgut after blood meal digestion.

## Conclusion

Leishmania parasites develop to a transmissible and infective form entirely within the confines of the alimentary tract of the sand fly, in contrast to numerous other arthropod-borne pathogens. We wished to further investigate the response of the sand fly midgut tissues that are occurring in response to blood meal ingestion and interactions with Leishmania parasites. The previously reported extensive sequencing of whole sand fly *Lutzomyia longipalpis *ESTs provided a large overview of the transcripts present in this vector; however, it did not provide information regarding tissue specific transcripts, particularly from the sand fly midgut or information regarding the midgut molecules which may be transcribed in response to blood feeding and digestion or interact with the Leishmania parasite. In the present work, the production of five different cDNA libraries generated a large number of redundant tissue specific transcripts for analysis as well as provided the capability of a comparative analysis between these cDNA libraries. Several molecules were identified in this midgut-specific transcriptome that were not identified in the EST database of whole sand fly sequences, including *LuloKZL *and *LuloDEF*.

The present analysis of midgut tissue from *L. longipalpis *further increases our knowledge of the molecular events which occur throughout the adult lifecycle of the sand fly. In general, it appears that the midgut reverts, after complete digestion and excretion of the blood meal, to a state nearly mimicking the midgut of a sand fly that has only taken a sugar meal. Comparing data generated from the sugar fed and blood fed sand fly midguts resulted in comparable global changes found in the same analysis of the midgut of *P. papatasi *[6]. Microvillar proteins, digestive proteases and peritrophin molecules are some of the transcripts identified as differentially represented between cDNA libraries when comparing unfed and blood fed sand flies. Interestingly, many molecules, such as microvillar proteins and digestive proteases, were found to be over or under represented when comparing the blood fed with the blood fed Leishmania-infected cDNA libraries. Similar results were observed in the midgut of *P. papatasi *when infected with *L. major*. This not only demonstrates the reproducibility of this technique of analyzing transcript abundance across cDNA libraries, but the redundancy present in the biology of blood feeding and digestion in sand flies as well as the Leishmania-vector interactions occurring between Old World and New World sand fly species. When comparing the uninfected and *L. infantum chagasi*-infected post blood meal digestion library we were astounded by the scarcity of differentially abundant transcripts when considering the number and volume of Leishmania parasites present in the midgut at the time points encompassed by the cDNA library. This data suggest the Leishmania parasite affects the midgut expression profile during the blood digestion process and not afterwards. It is likely that Leishmania parasite modulates the expression profile of other molecules but our approach was not able to detect these proteins probably for their low abundance.

Further testing employing more direct techniques such as real time PCR or other expression profiles approaches are still required to test the hypothesis that *L. infantum chagasi *is altering the expression of specific gut transcripts from the sand fly *Lutzomyia longipalpis*. However, the information presented on the current work and previous work on P. papatasi and *L. major *strongly suggest that Leishmania parasites can alter the expression of midgut transcripts that may be relevant for the survival and establishment of the parasite in the gut of the fly and that these changes may be occurring during the digestion of the blood meal and not afterwards.

## Methods

### Sand flies

*Lutzomyia longipalpis *sand flies (Jacobina strain) were maintained at the Laboratory of Malaria and Vector Research at the National Institute of Allergy and Infectious Diseases. Three to four-day post eclosion sand flies were allowed a 20% sucrose solution (sugar fed/unfed) or fed blood on anesthetized BALB/c mice (blood fed).

### Leishmania and sand fly infection

*L. longipalpis *sand flies infected by an artificial blood meal containing *Leishmania infantum chagasi*-infected macrophages (blood fed, infected) was based on the work of Tesh and Modi [16]. Briefly, *L. infantum chagasi *MHOM/BROO/MER/Strain 2 promastigote cultures were maintained in M199 (Sigma-Aldrich, St. Louis, MO) containing 20% (V\V) fetal bovine serum(FBS) (Invitrogen, Carlsbad, CA) and 100 units/ml Penicillin, 100 μg/ml Streptomycin, and 0.292 mg/ml Glutamine (PSG) (Invitrogen, Carlsbad, CA) at 25°C. Macrophage cell line J774A.1 (American Type Culture Collection, Manassas, VA) was cultured in RPMI (Invitrogen, Carlsbad, CA) containing 10% FBS and PSG at 37.0°C, 95% air, 5% CO2. At confluency, the macrophages were scraped from the culture flask and washed twice by centrifugation in phosphate buffered saline (PBS) at 380 × g for 10 minutes before resuspension in culture media. The washed macrophages were then placed in 5 wells of a 24-well culture plate at a concentration of 2 × 10^6 ^cells/ml and allowed to adhere for 90 minutes at 37°C, 5% CO_2_. Stationary-phase *L. infantum chagasi *culture was washed by centrifugation in PBS at 1200 × g for 15 minutes and resuspended in macrophage culture media. Nonadherent macrophages were removed by the replacement of the culture media and *Leishmania *parasites in macrophage added at a 5:1 ratio of parasite to macrophage. The parasites were co-cultured with the macrophages for 5 hours at 26°C. The culture was then washed to remove extracellular parasites and the macrophages scraped from the wells. Macrophages were confirmed to contain intracellular amastigotes by staining with QUICK III (Astral Diagnostics, Inc., West Deptford, NJ) according to the manufacture's protocol and visualized by light microscopy. The infected macrophage culture was centrifuged at 380 × g for 10 minutes and resuspended in 500 μl fresh whole mouse blood collected in heparin. The blood containing amastigote-infected macrophages were used for artificial blood feeding of sand flies as described [17].

### cDNA library construction

The conditions of the *Lutzomyia longipalpis *midguts harvested for the construction of the five cDNA libraries included: unfed/sugar fed four-day post-eclosion female midguts; midguts containing blood 1, 2, and 3 days post blood meal from female flies allowed to feed on BALB/c mice; midguts containing blood 1, 2, and 3 days post blood meal from female flies allowed an artificial blood meal containing *L. infantum chagasi *infected macrophages; midguts devoid of blood 5, 6, and 7 days post blood meal from gravid flies allowed to feed on BALB/c mice; midguts devoid of blood 5, 6, and 7 days post blood meal from gravid flies allowed an artificial blood meal containing *L. infantum chagasi *infected macrophages. All midguts used for the construction of cDNA libraries containing infected sand fly midguts were verified by microscopy as carrying Leishmania parasite infections comparable to mature infections seen in sand flies that are used routinely in transmission experiments. *L. longipalpis *midguts were dissected in phosphate buffered saline (PBS) and placed in RNAlater (Sigma-Aldrich, St. Louis, MO) and stored at 4°C prior to cDNA library construction. Libraries constructed using midguts at different time points consisted of two midguts at each day the midguts were dissected. *L. longipalpis *midgut mRNA was isolated from six midguts using the MicroFastTrack mRNA isolation kit (Invitrogen, San Diego, CA). The cDNA libraries were constructed using the SMART cDNA Library Construction Kit (Clontech, Mountain View, CA) as described previously [18].

### DNA Sequencing

Phage plaques lacking β-galactosidase activity were picked from the soft top agar using a sterilized wooden stick and placed into 75 μl of ultrapure water in a 96-well v-bottom plate. PCR was used to amplify the cDNA insert from 3 μl of the phage in water using FastStart PCR Master premixed PCR reagent (Roche Applied Science, Indianapolis, IN) and primers PT2F1 (AAGTACTCTAGCAATTGTGAGC) and PT2R1 (CTCTTCGCTATTACGCCAGCTG). Reaction conditions were 75°C, 3 min; 94°C, 4 min; 33 cycles of 94°C, 1 min; 49°C, 1 min; 72°C 2 min; a final extension of 72°C for 7 minutes. The PCR products were cleaned of buffering salts, dNTPs, and primers using ExcelaPure 96-well UF PCR purification plates (Edge Biosystems, Gaithersburg, MD) using three washes of 100 μl of ultrapure water and recovery in 30 μl of ultrapure water. Cycle sequencing was accomplished using in a reaction using BigDye Terminator v3.1 (Applied Biosystems, Foster City, CA), primer PT2F3 (TCTCGGGAAGCGCGCCATTGT), and 5 μl of the cleaned PCR product. The cycle sequencing products were prepared for sequencing by centrifugation through hydrated Sephadex G-50 (Amersham, Piscataway, NJ), desiccation, and rehydration with 10 μl sequencing buffer. Sequencing was performed using a 3730xl DNA analyzer (Applied Biosystems, Foster City, CA).

### Bioinformatics

Detailed reports of the bioinformatic analysis of the data are previously reported [19,20]. Succinctly, high N (unidentified nucleotide) content was removed at the 5' and 3' ends of each sequence any primer and vector nucleotides removed. Sequences from all five libraries were combined and contigs constructed from the clustering of homologous sequences based on 100% identity over 64 nucleotides while sequences with greater than 5% N's were discarded. Three frame translated sequences were supplied to the appropriate BLAST algorithm for comparison to the contents of the NCBI non-redundant protein database, the Gene Ontology database [21], the conserved domain database [22] which contains the eukaryotic clusters of orthologous groups (COG), Simple Modular Architecture Tool (SMART) and Protein Family Database (Pfam)[23,24]. Customized databases of mitochondrial and ribosomal RNA nucleotide sequences were also used for the comparison of cDNA sequences. The predicted presence of a signal secretion peptide or transmembrane helices was determined using the SignalP [25]or TMHMM server [26], respectively. A custom program, Count Libraries, was used to identify the number of transcripts that each library contributed to the formation of a contig (JMC Ribeiro). The contigs, information regarding each contig, the BLAST and SignalP results were combined in a hyperlinked Excel spreadsheet and each contig annotated by manually assigning the most likely predicted function based on BLAST results. Sequences were aligned using Clustal X, version 1.83, and converted to graphical aligned sequences using BioEdit, version 7.0.5.3[27]. Phylogenetic analysis was conducted on amino acid alignments using TREE-PUZZLE, version 5.2, generating trees by maximum likelihood using quartet puzzling with 10,000 puzzling steps to calculate node support [28]. Statistical significance in the number of transcripts per cluster within that same cluster, between cDNA libraries, was analyzed using Pearson's Chi-square test.

## Authors' contributions

RCJ participated in the conception and coordination of the study, infection of the sand flies, construction of cDNA libraries, sequencing of the transcripts from the cDNA libraries, bioinformatics analysis and annotation, sequence alignment, phylogenetic analysis and drafting the manuscript. CRT participated in the infection of the sand flies. DE provided uninfected sand flies and helped in sand fly infection. AL participated in sand fly rearing and infection. JM participated in the sequencing of the transcripts from the cDNA libraries. FO participated in the sequencing of the transcripts from the cDNA libraries. RBG participated in the infection of the sand flies. JGV participated in the conception and coordination of the study and drafting the manuscript.
